# The evaluation of risk factors for prolonged viral shedding during anti-SARS-CoV-2 monoclonal antibodies and long-term administration of antivirals in COVID-19 patients with B-cell lymphoma treated by anti-CD20 antibody

**DOI:** 10.1186/s12879-024-09631-3

**Published:** 2024-07-22

**Authors:** Shuhei Maruyama, Daiki Wada, Shuji Kanayama, Haruka Shimazu, Yumiko Miyano, Akira Inoue, Masami Kashihara, Kazuyuki Okuda, Fukuki Saito, Yasushi Nakamori, Kazuyoshi Ishii, Yasuyuki Kuwagata

**Affiliations:** 1 Department of Emergency and Critical Care Medicine, Kansai Medical University General Medical Center, 10-15 Fumizono-cho, Moriguchi, Osaka 570-8507 Japan; 2 Department of Hematology and Oncology, Kansai Medical University General Medical Center, 10-15 Fumizono-cho, Moriguchi, Osaka 570-8507 Japan; 3 Department of Emergency and Critical Care Medicine, Kansai Medical University Hospital, 2-3-1 Shinmachi, Hirakata, Osaka 573-1191 Japan

**Keywords:** COVID-19, B-cell lymphoma, Persistent infection, Anti-CD20, Bendamustine

## Abstract

**Background:**

The global impact of the coronavirus disease 2019 (COVID-19) pandemic has resulted in significant morbidity and mortality. Immunocompromised patients, particularly those treated for B-cell lymphoma, have shown an increased risk of persistent infection with SARS-CoV-2 and severe outcomes and mortality. Multi-mutational SARS-CoV-2 variants can arise during the course of such persistent cases of COVID-19. No optimal, decisive strategy is currently available for patients with persistent infection that allows clinicians to sustain viral clearance, determine optimal timing to stop treatment, and prevent virus reactivation. We introduced a novel treatment combining antivirals, neutralizing antibodies, and genomic analysis with frequent monitoring of spike-specific antibody and viral load for immunocompromised patients with persistent COVID-19 infection. The aim of this retrospective study was to report and evaluate the efficacy of our novel treatment for immunocompromised B-cell lymphoma patients with persistent COVID-19 infection.

**Methods:**

This retrospective descriptive analysis had no controls. Patients with B-cell lymphoma previously receiving immunotherapy including anti-CD20 antibodies, diagnosed as having COVID-19 infection, and treated in our hospital after January 2022 were included. We selected anti-SARS-CoV-2 monoclonal antibodies according to subvariants. Every 5 days, viral load was tested by RT-PCR, with antivirals continued until viral shedding was confirmed. Primary outcome was virus elimination. Independent predictors of prolonged viral shedding time were determined by multivariate Cox regression.

**Results:**

Forty-four patients were included in this study. Thirty-five patients received rituximab, 19 obinutuzumab, and 26 bendamustine. Median treatment duration was 10 (IQR, 10–20) days; 22 patients received combination antiviral therapy. COVID-19 was severe in 16 patients, and critical in 2. All patients survived, with viral shedding confirmed at median 28 (IQR, 19–38) days. Bendamustine use or within 1 year of last treatment for B-cell lymphoma, and multiple treatment lines for B-cell lymphoma significantly prolonged time to viral shedding.

**Conclusions:**

Among 44 consecutive patients treated, anti-SARS-CoV-2 monoclonal antibodies and long-term administration of antiviral drugs, switching, and combination therapy resulted in virus elimination and 100% survival. Bendamustine use, within 1 year of last treatment for B-cell lymphoma, and multiple treatment lines for B-cell lymphoma were the significant independent predictors of prolonged viral shedding time.

**Supplementary Information:**

The online version contains supplementary material available at 10.1186/s12879-024-09631-3.

## Background

Coronavirus disease 2019 (COVID-19) has currently infected over 600 million people worldwide and caused over 6 million deaths. Mutations in the spike protein of severe acute respiratory syndrome coronavirus 2 (SARS-CoV-2) that escape from neutralizing antibodies can arise in immunocompromised patients with prolonged infection. Higher mortality after COVID-19 is a developing issue among patients with non-Hodgkin lymphoma treated with B-cell depleting immunotherapy [1]. Several studies have reported that COVID-19 infection is associated with severe disease and high mortality in patients with depleted B cells [2, 3]. Thanks to high-quality randomized trials, management of patients with SARS-CoV-2 infection has developed rapidly over the past year. Although these trials have justifiably focused on preventing severe disease in the majority of patients, their benefits might not apply to immunodeficient patients at high risk for recurrence of persistent infection. Although rapid viral decrease with normal combined antiviral and antibody-based therapy might preclude further evolution, no optimal, decisive strategy is currently available for patients with persistent infection that allows clinicians to sustain viral clearance, determine optimal timing to stop treatment, and prevent virus reactivation.

From September 2021, we introduced novel treatment combining antiviral and neutralizing antibody-based therapies with monitoring of spike-specific antibody and viral load for immunocompromised patients with persistent COVID-19 infection [4]. In particular, it was important to elucidate the efficacy of this treatment on B-cell lymphoma patients suffering B-cell depletion due to anti-CD20 monoclonal antibody treatment. For refractory cases in particular, genomic analysis was performed, and data obtained about drug resistance mutations were used as a reference to determine which antiviral drugs and antibody therapies might be effective to treat these patients [5].

This study aimed to report the efficacy and safety of novel treatment combining antiviral and neutralizing antibody-based therapies with monitoring of spike-specific antibody and viral load for immunocompromised B-cell lymphoma patients with persistent COVID-19 infection.

## Methods

### Study design and participants

**Fig. 1:**
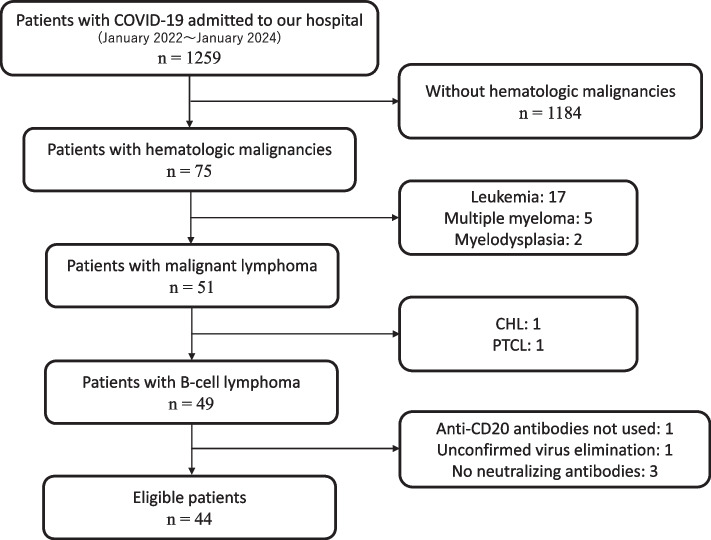
Flow of the patients with COVID-19 in our hospitals. CHL, classical Hodgkin lymphoma; PTCL, peripheral T-cell lymphoma

This retrospective, single-center, descriptive study had no controls. Subjects were patients with B-cell lymphoma previously receiving immunotherapy including anti-CD20 antibodies and diagnosed as having COVID-19 confirmed by reverse transcription polymerase chain reaction (RT-PCR) test for SARS-CoV-2 from nasopharyngeal swab and treated in the Department of Emergency and Critical Care Medicine, Kansai Medical University General Medical Center, Osaka, Japan, between January 2022 and January 2024. All patients were treated according to our protocol targeting viral shedding, which was finally confirmed by RT-PCR. Patients whose follow-up was terminated without confirmation of viral shedding by RT-PCR were excluded, as were patients in whom protocols regarding treatment for COVID-19 were not followed (Fig. 1).

### Treatment protocol for patients with COVID-19

Samples from patients diagnosed as having COVID-19 were subjected to single nucleotide polymorphism PCR assays or whole-genome analysis to identify subvariants.　We selected anti-SARS-CoV-2 monoclonal antibodies according to the subvariants with reference to previous reports [6–9]. Treatment with sotrovimab was selected for BA.1, imdevimab/casirivimab for BA.2, imdevimab/casirivimab or tixagevimab/cilgavimab for BA.5, and sotrovimab for the XBB lineage.

Until oral antivirals were approved in Japan, remdesivir was the only option. Following their approval, antivirals were selected by each physician from among remdesivir, molnupiravir, nirmatrelvir/ritonavir, and ensitrelvir, based on renal or liver function, concomitant medications, and the patient’s ability to take them orally. If persistent viral infection was empirically anticipated, a combination of two antiviral drugs was also administered. Every 5 days, the viral load was tested by RT-PCR, and antivirals were continued until viral shedding was confirmed. Viral shedding was defined as viral load below 1 copy/µL. Because we experienced a rebound in viral load after the viral load was below 1 copy/µL, we continued to measure viral load thereafter. When antiviral drugs were judged to be ineffective, they were changed or added to.

When viral load did not decrease after administration of neutralizing antibodies and antiviral drugs, whole-genome analysis was performed. When drug-resistant mutations reported in past papers and drug company fact sheets were found, the drug was changed based on these results.

### Measurement of spike-specific and nucleocapsid protein antibodies

Anti SARS-CoV-2 spike-specific antibody and nucleocapsid protein antibody were measured by electrochemiluminescence immunoassay using Elecsys Anti-SARS-CoV-2 (Roche Diagnostics). Anti-spike-specific antibody values > 0.8 U/mL and anti-nucleocapsid protein antibody > 1.0 U/mL were considered positive per manufacturer’s information.

### Measurement of viral load and identification of subvariants

After RNA extraction, SARS-CoV-2 was detected by RT-PCR with SARS-CoV-2 Detection Kit -Multi- (Toyobo, Osaka, Japan), on a QuantStudio 5 real-time PCR system (Thermo Fisher Scientific, MA, USA) according to manufacturer’s instructions. The viral load of each sample was quantified based on a standard curve by including a preparation of standard material with a known nucleic acid concentration in the RT-PCR analysis. Variant-specific single nucleotide polymorphisms were identified by RT-PCR according to the prevalent variants by using variant-specific probes and primers for ins214EPE (Takara Bio Co., Shiga, Japan) and L452R, G339D, E484K and N460K (Thermo Fisher Scientific, MA, USA).

### Genome analysis methods

If the viral load of the initial specimen was > 50 copies/µL, whole-genome analysis was performed on the initial sample to identify subvariants. When viral load did not decrease after treatment, subsequent genomic analysis was performed. The sequencing run was performed with Ion AmpliSeq SARS-CoV-2 Insight Research Assay on an Ion Torrent Genexus Integrated Sequencer (Thermo Fisher Scientific).

### Data collection

For each patient, data were obtained on their demographics, classification of B-cell lymphomas, diagnosis and treatment history for B-cell lymphoma, initial laboratory results, and COVID-19-related data including vaccination frequency, viral load, spike-specific antibody, nucleocapsid protein antibody, subvariants, COVID-19 severity, presence of CT findings suggestive of COVID-19 pneumonia, time from onset of COVID-19 to viral elimination, and the antiviral, neutralizing antibody, corticosteroid, and anti-inflammatory drugs administered.

### Statistical analysis and ethical concerns

Categorical data are summarized as frequencies and proportion, and continuous variables are as shown as the median and interquartile 25–75th percentile range (IQR). To identify factors prolonging time to viral shedding, univariate analysis was first conducted using the Kaplan-Meier method. In the multivariate Cox regression analysis to determine independent predictors, six variables with *p* < 0.05 in the univariate analysis were selected to avoid overfitting. Statistical analysis was performed with SPSS 28.0 software (IBM Corp, USA).

This study was conducted according to the principles expressed in the Declaration of Helsinki and approved by the Institutional Review Board of Kansai Medical University General Medical Center (Study Number: 2022300), which waived the requirement for written informed consent due to the retrospective study design.

## Results

### Study subjects

During the study period, 1259 patients with COVID-19 were treated at our hospital. Among them, 49 patients had been previously treated for B-cell lymphoma. Five cases were excluded for respective reasons, 44 patients met the study’s entry criteria (Fig. 1). Detailed patient information is presented in Supplementary Tables 1 and 2, with patient numbers ordered by date of admission.

### B-cell lymphoma

The majority of lymphoma subtypes were follicular lymphoma (*n* = 20, 45.5%) and diffuse large B-cell lymphoma (DLBCL) (*n* = 17, 38.6%). The time between last immunosuppressive treatment and SARS-COV-2 infection was 3.5 (IQR, 1–15) months. Rituximab was administered in 35 (79.5%) cases, obinutuzumab in 19 (43.2%), and bendamustine in 26 (59.1%) (Table 1, Supplementary Table 1). These immunosuppressive agents were used in combinations rather than as single agents.

**Table 1:** Patient characteristics, information on B-cell lymphoma, initial laboratory results, and initial information related to SARS-CoV-2

Factor	Value
Age, years, (range)	68 (56–79)
Male sex, n (%)	23 (52.3)
**Information on B-cell lymphoma**	
Period since diagnosis of B-cell lymphoma to SARS-CoV-2 onset, months, (range)	27 (14–83)
Period since last treatment for B-cell lymphoma to SARS-COV-2 onset, months, (range)	3.5 (1–15)
Subtype of B-cell lymphomas, n (%)	
Follicular lymphoma	20 (45.5)
Diffuse large B-cell lymphoma	17 (38.6)
Lymphoplasmacytic lymphoma	2 (4.5)
Mucosa-associated lymphoid tissue lymphoma	2 (4.5)
Mantle cell lymphoma	1 (2.3)
B-lymphoblastic lymphoma	1 (2.3)
Other B-cell non-Hodgkin lymphoma	1 (2.3)
Number of patients using anti-CD20 medication, n (%)	
Rituximab	35 (79.5)
Obinutuzumab	19 (43.2)
Number of patients using other medication for lymphoma, n (%)	
Bendamustine	26 (59.1)
Polatuzumab vedotin	9 (20.5)
Within 1 year of last treatment for B-cell lymphoma, n (%)	31 (70.5)
Multiple treatment lines, n (%)	13 (29.5)
**Initial laboratory results**	
Absolute lymphocyte count, cells/mL, (range)	482 (268–800)
Absolute neutrophil count, cells/mL, (range)	2592 (1550–4072)
Platelet count, cells/mL, (range)	166,000 (78,000–198000)
Hemoglobin, g/dL, (range)	11.9 (9.6–13.7)
C-reactive protein, mg/dL, (range)	2.8 (0.9–9.3)
Lactate dehydrogenase, U/L, (range)	274 (190–366)
Interleukin-6, pg/mL, (range)	27.2 (8.3–68.7)
**Initial information related to SARS-CoV-2**	
≥ 3 COVID-19 vaccinations, n (%)	29 (65.9)
Period since onset of COVID-19 to admission to our hospital, days, (range)	5.5 (1.75–14.25)
Transferred from another hospital with a diagnosis of persistent infection, n (%)	6 (13.6)
Positive spike-specific antibody, n (%)	33 (75.0)
Positive nucleocapsid protein antibody, n (%)	3 (6.8)
Viral load, copies/µL, (range)	68,026 (3704–1016644)
CT findings suggestive of COVID-19 pneumonia	28 (63.6)
Sublineages of omicron variant, n (%)	
BA.1	5 (11.4)
BA.2 (including FR)	11 (25.0)
BA.5 (including BF and BU)	18 (40.9)
XBB (including EG)	7 (15.9)
BA.2.86	1 (2.3)
Not assessed	2 (4.5)

### COVID-19

Twenty-nine patients (65.9%) had received three or more SARS-CoV-2 vaccinations, six received prophylactic tixagevimab/cilgavimab, and 33 (75.0%) patients were initially seropositive. Viral genomic analysis was performed on 42 cases, excluding two with low initial viral load, with the highest number of 18 (40.9%) cases being of BA.5 lineage. Anti-SARS-CoV-2 monoclonal antibodies were administered to all patients with reference to the prevalent variants and the results of single nucleotide polymorphism PCR assays (Supplementary Tables 4), with multiple anti-SARS-CoV-2 monoclonal antibodies administered in seven cases based on genomic analysis results. The median duration of antiviral treatment was 10 (IQR, 10–20) days, with 22 (50.0%) patients receiving combination therapy and 27 (61.4%) changing regimens during the treatment course. Oxygen or invasive ventilation was required during the disease course in 18 (40.9%) patients, and corticosteroids were administered in 18 (40.9%). All patients survived, with viral shedding confirmed at a median of 28 (IQR, 19–38) days. Nucleocapsid protein antibody measured at viral shedding was positive in 8 (18.2%) cases (Tables 1 and 2, Supplementary Tables 2 and 3).

**Table 2:** Treatment, severity, and outcome of COVID-19

Factor	Value
Patients using neutralizing antibody, n (%)	
Sotrovimab	24 (54.5)
Imdevimab/casirivimab	22 (50.0)
Tixagevimab/cilgavimab (treatment)	4 (9.1)
Tixagevimab/cilgavimab (prophylaxis)	6 (13.6)
Patients using antiviral, n (%)	
Remdesivir	24 (54.5)
Molnupiravir	25 (56.8)
Nirmatrelvir/ritonavir	23 (52.3)
Ensitrelvir	17 (38.6)
Combination therapy (2 antivirals)	22 (50.0)
First treated with combination therapy	9 (20.5)
Multiple antiviral regimens	27 (61.4)
Duration of antiviral, days, (range)	10 (10–20)
Patients using corticosteroid, n (%)	18 (40.9)
Patients using baricitinib, n (%)	8 (18.2)
Period since onset to viral shedding, days, (range)	28 (19–38)
Positive nucleocapsid protein antibody at viral shedding, n (%)	8 (18.2)
Severity of patients with COVID-19, n (%)	
Moderate	26 (59.1)
Severe	16 (36.4)
Critical	2 (4.5)
Mortality, n (%)	0 (0)

### Factors prolonging time to viral shedding

**Fig. 2:**
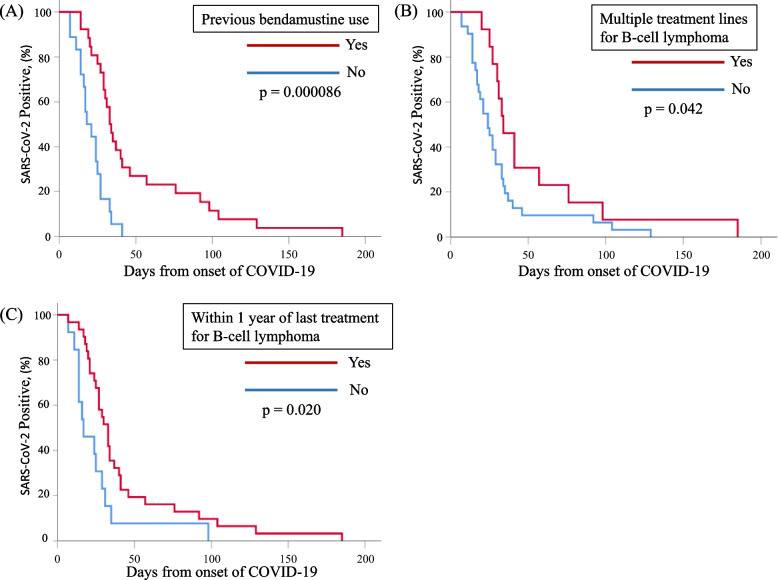
Kaplan-Meier survival curves between COVID-19 onset and viral shedding based on previous bendamustine use (**A**), multiple treatment lines for B-cell lymphoma (**B**), and within 1 year of last treatment for B-cell lymphoma (**C**)

In univariate analysis, time to viral disappearance was significantly prolonged in patients with a history of bendamustine use (*p* < 0.001) within 1 year of the last immunosuppressive treatment (*p* = 0.020), multiple treatment lines for B-cell lymphoma (*p* = 0.042), initial absolute lymphocyte count ≦482 (median value) cells/mL (*p* = 0.014), remdesivir use (*p* = 0.004), corticosteroid use (*p* = 0.008), and severe or critical COVID-19 (*p* = 0.007), and was significantly shorter with subtype DLBCL (*p* = 0.005) (Table 3). In the multivariate analysis to determine independent predictors, six variables with *p* < 0.05 in the univariate analysis were selected to avoid overfitting. Bendamustine use (hazard ratio [HR] 3.056, 95% confidence interval [CI] 1.238–7.541, *p* = 0.015), within 1 year of last treatment for B-cell lymphoma (HR 2.373, 95% CI 1.141–4.932, *p* = 0.021), and multiple treatment lines for B cell lymphoma (HR 2.601, 95% CI 1.102–6.138, *p* = 0.029) significantly prolonged time to viral shedding (Table 3, Fig. 2).

**Table 3:** Kaplan-Meier method and multivariate Cox regression analysis to identify risk factors of prolonged viral shedding time

Variables	Univariate analysis		Multivariate analysis		
	χ2	*p* Value	*p* Value	Hazard ratio	95% Confidence interval
Age (≥ 70 years)	1.356	0.244			
Gender (Male)	0.177	0.674			
Treatment for B-cell lymphoma					
Rituximab	0.003	0.954			
Obinutuzumab	1.829	0.176			
Bendamustine	15.422	< 0.001	0.015	3.056	1.238–7.541
Polatuzumab vedotin	0.324	0.570			
Within 1 year of last treatment for B-cell lymphoma	5.410	0.020	0.021	2.373	1.141–4.932
Multiple treatment lines	4.150	0.042	0.029	2.601	1.102–6.138
Subtype of malignant lymphomas					
Follicular lymphoma	1.575	0.209			
Diffuse large B-cell lymphoma	7.750	0.005			
Less than 2 vaccinations	0.006	0.939			
Negative spike-specific antibody (seronegative)	0.279	0.597			
Absolute lymphocyte count ≦482 cells/mL	6.047	0.014	0.909	0.954	0.427–2.133
Treatment for COVID-19					
Remdesivir	8.204	0.004	0.372	0.708	0.332–1.511
Molnupiravir	3.769	0.052			
Nirmatrelvir/ritonavir	1.683	0.195			
Ensitrelvir	0.055	0.814			
First treated with combination therapy	0.427	0.513			
Corticosteroid	7.099	0.008	0.093	1.898	0.899–4.008
Baricitinib	2.279	0.131			
Severe or critical COVID-19	7.312	0.007			
CT findings suggestive of COVID-19 pneumonia	3.326	0.068

## Discussion

In patients with B-cell immunodeficiency, infection with SARS-CoV-2 is prolonged and prognosis is poor. We reported the administration of monoclonal antibodies and antiviral drugs to patients with malignant lymphoma and post-organ transplantation, using viral and anti-spike specific antibody levels as indicators [4]. For cases with persistent or rebounding viral load, we have performed whole-genome analysis of SARS-CoV-2 to confirm presence or absence of drug resistance gene mutations for use as a reference for drug selection [5]. In the present study, we focused on 44 cases of B-cell lymphoma treated with anti-CD20 antibodies to retrospectively examine the efficacy of our therapeutic strategy and confirmed viral shedding and survival in all cases.

Anti-SARS-CoV-2 monoclonal antibodies have been trialed in patients with hematological malignancies with prolonged SARS-CoV-2 shedding even before the omicron variant emerged [10, 11]. Assanto et al. reported that anti-SARS-CoV-2 monoclonal antibodies reduced mortality in lymphoma patients during the epidemic of BA.1 susceptible to sotrovimab [12]. After emergence of the BA.2 sub-variant with reduced neutralizing activity of anti-SARS-CoV-2 monoclonal antibodies, the same effect cannot be expected for neutralizing antibodies. We used single nucleotide polymorphism PCR assays or whole-genome analysis to determine the subvariants, which were used as reference for the selection of anti-SARS-CoV-2 monoclonal antibodies [6–9]. Between January 2022 and January 2024, the subvariants prevalent in Japan changed from the delta variant to the omicron variants BA.1, BA.2, BA.5, XBB.1.5, XBB.1.9, XBB.1.16, EG.5, and BA.2.86. During this transition, the identification of subvariants was important in the selection of anti-SARS-CoV-2 monoclonal antibodies. Whole-genome analysis is accurate but time-consuming, so single nucleotide polymorphism PCR assays, which give results in 2 h, were performed on most cases before treatment. The L452R mutation was targeted to differentiate the delta variant from BA.1; ins214EPE in BA.1 and BA.2; L452R and G339D in BA.2 and BA.5; and the N460K mutation in BA.5 and XBB [13–15]. Since emergence of the XBB subvariant, sotrovimab has shown some residual neutralizing activity and is believed effective against SARS-COV-2 due to its Fc-dependent effector functions such as antibody-dependent cellular cytotoxicity [16, 17].

In the early stages of this cohort, remdesivir was the only antiviral drug approved in Japan against SARS-CoV-2, so the only option was to continue it. Subsequently, molnupiravir and nirmatrelvir/ritonavir were approved, and we switched to these antivirals for cases in which remdesivir did not reduce viral load. We performed additional whole-genome analysis in cases with rebound viral load after long-term treatment with remdesivir and found genetic mutations in the non-structural protein (NSP) 12 region with reported drug resistance, including C799Y and V166A [5, 18] (Supplementary Table 3). These experiences have led to avoidance of long-term use of the same regimen, changing regimens in the short term, or using 3CL-protease inhibitor in combination with RNA-dependent RNA polymerase inhibitor. The use of molnupiravir increased because of no reports of associated drug resistance mutations [19, 20]. Although drug resistance mutations are reported in in-vitro data for nirmatrelvir/ritonavir, no drug resistance mutations were observed in our cohort [21–23]. In 2023, the 3CL-protease inhibitor ensitrelvir was approved only in Japan, increasing its use. However, Kiso et al. reported a M49L mutation in the NSP5 region as a drug resistance mutation that requires attention after treatment with ensitrelvir [24]. We also found M49L mutations in three patients and M49I mutations in one patient after ensitrelvir treatment. This led us to avoid long-term treatment with ensitrelvir (Supplementary Table 3).

Several reports on combination drug therapy in patients with hematological malignancies have emerged from Italy in recent years. Pasquini et al. used remdesivir and nirmatrelvir/ritonavir combination therapy in 14. Most of their eligible patients had non-Hodgkin lymphoma, with a median of 42 days from SARS-CoV-2 infection and a persistent infection with previous treatment in 57% of cases. Viral clearance was confirmed for all participants after 5–22 days of combination therapy [25]. We also treated 22 of our patients with two different antiviral drugs. However, the initial regimen was combination therapy in nine cases, and combination therapy was introduced in 13 cases because the virus did not decrease with a single medication. In the group first treated with combination therapy, the duration of antivirals ranged from 5–20 (median, 10) days and no significant differences. In our cohort, we were unable to demonstrate the effectiveness of combination therapies. Mikulska et al. used remdesivir and nirmatrelvir/ritonavir or molnupiravir plus monoclonal antibodies in 22 patients with persistent SARS-CoV-2 infection. Antiviral were administered for 10 days, with viral clearance rates of 75% on day 14 and 73% on day 30. Four patients received a second course of treatment, but three died [26]. The results of these two trials and our data suggest that combination antiviral therapy, plus anti-SARS-CoV-2 monoclonal antibodies, may be effective against persistent SARS-CoV-2 infection in immunocompetent patients, but the 10-day treatment period may be too short.

Nucleocapsid protein antibody usually positive 2–3 weeks after infection measured at the time of viral shedding in this study was positive in only 8 (18.2%) cases [27]. In three cases, nucleocapsid antibodies were positive before treatment, and in two recent cases, nucleocapsid antibody titers increased immediately after immunoglobulin preparations were administered for hypogammaglobulinemia. In these two cases, it is probable that the immunoglobulin preparations contained nucleocapsid antibodies [28]. It is telling that the viral shedding was achieved not by the patient’s immunity but by the effect of the anti-SARS-CoV-2 monoclonal antibodies and long-term antiviral treatment. We consider it reasonable that Pasquini et al. [25] and our protocol set the termination of treatment as viral shedding, which we confirmed in all cases. The median duration of antiviral treatment was 10 days but exceeded 30 days in nine cases.

In this study, factors leading to persistent infection were investigated in B-cell lymphoma after treatment with anti-CD20 drugs. Many reports exist of persistent infection with SARS-CoV-2 in patients with B-cell lymphoma previously treated with rituximab or obinutuzumab [29, 30]. To our knowledge, this is the first report to investigate risk factors for persistent infection with SARS-CoV-2 restricted to B-cell lymphomas in patients with a history of anti-CD20 treatment. In univariate analysis, time to viral shedding was significantly prolonged by factors such as history of bendamustine use, corticosteroid use or remdesivir use, within 1 year of the last treatment or multiple treatment lines for B-cell lymphoma, initial low absolute lymphocyte count, and severe or critical COVID-19. The DLBCL subtype of B-cell lymphoma significantly reduced the time to viral shedding. Some of these factors were expected based on clinical experience and previous reports [1, 31]. Univariate analysis showed that the use of remdesivir significantly prolonged the time to viral shedding. This may be due to the longer duration of treatment in the six patients who received intermittent and long administration of remdesivir at other hospitals but were transferred to our hospital with persistent infection. Furthermore, multivariate Cox regression showed significant differences for a history of bendamustine use, within 1 year of last treatment for B-cell lymphoma, and multiple lines of treatment for B-cell lymphoma. A history of bendamustine use, which had the highest HR, has been reported to increase mortality in COVID-19 and cause poor serologic response after vaccination [12, 32]. Ichikawa et al. reported that in patients with malignant lymphoma and a history of bendamustine and anti-CD20 use, causes of prolonged viral shedding are CD4+ T-cell dysfunction and low neutralization activity [33]. An Italian cohort study of 856 cases of malignant lymphoma with COIVD-19 noted age, gender, lymphocyte count and platelet count as factors associated with risk of death. The study was conducted in the early stages of the pandemic, with a 100-day mortality rate of 23% [34]. Our study also assessed the impact of these variables on the duration of viral shedding. However, these variables were not significantly associated with time to viral shedding. One possible difference could be the difference in treatment strategy, which included anti-SARS-CoV-2 monoclonal antibodies and long-term administration of antivirals for viral shedding with genomic analysis in our study, whereas the Italian cohort study in the literature used the usual COVID-19 treatment.

We have performed whole-genome analyses to search for drug resistance gene mutations in cases of prolonged SARS-CoV-2 infection. Some cases from this cohort were also reported in previous papers [5]. In a small population, drug resistance gene mutations were found against anti-SARS-CoV-2 monoclonal antibodies in seven cases, against remdesivir in two cases, and against ensitrelvir in four cases (Supplementary Table 3). No drug resistance gene mutations have been reported for molnupiravir, so it could not be identified. Notably, despite reports of in-vitro drug resistance gene mutations for nirmatrelvir/ritonavir [21–23], we found none in our cohort. Our search of articles revealed no reports of treatment of cases of persistent infection with SARS-CoV-2 with reference to repeated whole-genome analysis results. In-vitro studies during the SARS-CoV-2 pandemic reported that the virus mutated to escape the drug, but clinicians failed to widely translate this information into clinical practice. A medical system must be developed in which whole-genome analysis can be routinely performed in preparation for future viral pandemics.

The main limitation of this study is the lack of sufficient evidence to support the efficacy of these monoclonal antibodies and antiviral drugs administered in the treatment of patients infected with COVID-19. There are no trials or rigorous research that includes randomization and larger sample sizes to show the efficacy of neutralizing antibodies as a therapeutic agent. Nor are there many clinical reports on the application of genomic analysis results in the selection and determination of antiviral or neutralizing antibody drugs for patients with B-cell immunodeficiency and refractory SARS-CoV-2 infection. Despite our experience with cases in which anti-SARS-CoV-2 monoclonal antibodies and long-term administration of antiviral drugs, switching, and combination therapy were applied in the treatment of COVID-19, further research is needed to accurately determine the effectiveness of this strategy. We retrospectively evaluated the effect of antiviral and antibody treatment for SARS-CoV-2 infection in malignant lymphoma cases. Viral elimination was achieved in all cases, but the number of patients included our COVID-19 treatment strategy was relatively small to demonstrate efficacy of treatment. Determination of risk factors for delayed viral shedding are important to note and to improve treatment strategy in this subgroup. The treatment according to genomic evaluation is also interesting, although the combination treatment could not be shown to have significantly different results in our analysis.

## Conclusions

Among 44 consecutive patients treated, anti-SARS-CoV-2 monoclonal antibodies and long-term administration of antiviral drugs, switching, and combination therapy resulted in virus elimination and 100% survival. Bendamustine use, within 1 year of last treatment for B-cell lymphoma, and multiple treatment lines for B-cell lymphoma were the significant independent predictors of prolonged viral shedding time.

### Supplementary Information

Supplementary Material 1.

Supplementary Material 2.

Supplementary Material 3.

Supplementary Material 4.

## Data Availability

All relevant data are contained within the paper and its Supplementary Material files. The 13 SARS-CoV-2 strains sequences from 11 patients obtained in this study were submitted to the DDBJ (DNA Data Bank of Japan).
