# Supplementary material for: The evaluation of risk factors for prolonged viral shedding during anti-SARS-CoV-2 monoclonal antibodies and long-term administration of antivirals in COVID-19 patients with B-cell lymphoma treated by anti-CD20 antibody
Source: BMC Infect Dis. 2024 Jul 22;24:715. doi: 10.1186/s12879-024-09631-3 (PMC11265166; doi:10.1186/s12879-024-09631-3)
Supplement: Supplementary file 1 — Supplementary Material 1. [file 12879_2024_9631_MOESM1_ESM.docx]

| **Supplementary Table 1. Patient Characteristics, Information on Malignant Lymphoma, and Initial Information on SARS-CoV-2** | | | | | | | | | | | | |
| --- | --- | --- | --- | --- | --- | --- | --- | --- | --- | --- | --- | --- |
| No. | Age | Gender | | Subtype of malignant lymphoma | Treatment for B-cell lymphoma | Number of treatment lines | Months from B-cell lymphoma onset to SARS-CoV-2 infection | Months from last treatment for B-cell lymphoma to SARS-COV-2 infection | Vaccination frequency | Initial spike-specific antibody (U/ml) | Initial nucleocapsid protein antibody (U/ml) | Initial viral load (copies/µL) |
| 1 | 65 | | F | FL | GB, Gm | 1 | 19 | 2 | 2 | >0.4 | >0.1 | 19681 |
| 2 | 74 | | M | DLBCL | R-THP-COP | 1 | 5 | 1 | 2 | 13.9 | 0.12 | 494 |
| 3 | 81 | | F | FL | GB, Rm | 1 | 16 | 1 | 3 | >0.4 | >0.1 | 387834 |
| 4 | 74 | | M | FL | R-CVP, Rm, GB, Gm | 2 | 86 | 19 | 2 | >0.4 | >0.1 | 156023 |
| 5 | 51 | | M | FL | G-CHOP, Gm | 1 | 88 | 1 | 3 | >0.4 | >0.1 | 351189 |
| 6 | 79 | | F | FL | R-CHOP, RB, GB, Gm | 3 | 111 | 13 | 3 | 8945 | >0.1 | 4034 |
| 7 | 53 | | F | FL | GB, Gm | 1 | 37 | 14 | 3 | >0.4 | >0.1 | 39018 |
| 8 | 63 | | F | LPL | DRC, RB | 3 | 66 | 6 | 3 | >0.4 | >0.1 | 9378200 |
| 9 | 67 | | F | FL | GB, Gm | 1 | 45 | 4 | 3 | >0.4 | >0.1 | 443 |
| 10 | 79 | | F | FL | R-CVP, Rm | 1 | 127 | 107 | 3 | >0.4 | >0.1 | 59 |
| 11 | 56 | | F | DLBCL | R-CHOP | 1 | 8 | 4 | 3 | 11.7 | >0.1 | 4137555 |
| 12 | 80 | | F | DLBCL | R-CHOP, Pola-BR | 1 | 5 | 3 | 2 | 14.9 | 0.11 | 2839493 |
| 13 | 76 | | M | FL | R-CHOP, GB, Gm | 2 | 104 | 0 | 3 | >0.4 | >0.1 | 170731 |
| 14 | 70 | | M | DLBCL | R-CHOP | 1 | 11 | 6 | 2 | >0.4 | 0.11 | 857489 |
| 15 | 71 | | M | FL | GB, Rm | 1 | 12 | 2 | 2 | >0.4 | >0.1 | 2714 |
| 16 | 47 | | M | FL | GB, Gm | 1 | 12 | 3 | 2 | 6.48 | >0.1 | 21 |
| 17 | 54 | | M | FL | R-CHOP, RB, Rm, GB, Gm | 3 | 154 | 5 | 3 | >0.4 | >0.1 | 52051 |
| 18 | 81 | | M | FL | R, GB, Gm | 1 | 15 | 2 | 2 | 2814 | 0.33 | 537627 |
| 19 | 35 | | F | DLBCL | R-CHOP, ASCT | 1 | 132 | 120 | None | 452 | 5.11 | 2420183 |
| 20 | 68 | | M | DLBCL | THP-COP, Rm | 1 | 82 | 70 | 3 | 3688 | >0.1 | 29 |
| 21 | 67 | | M | DLBCL | R-CHOP | 1 | 46 | 40 | 3 | 1524 | >0.1 | 168841 |
| 22 | 49 | | M | LPL | R | 1 | 13 | 1 | 3 | 11816 | 0.11 | 446956 |
| 23 | 66 | | F | FL | GB, Gm | 1 | 28 | 6 | 3 | 3051 | >0.1 | 19544816 |
| 24 | 51 | | M | FL | R-CHOP, Rm | 1 | 17 | 1 | 4 | 2.02 | >0.1 | 2033 |
| 25 | 82 | | F | MALT | RB | 1 | 46 | 42 | 5 | >0.4 | >0.1 | 84001 |
| 26 | 78 | | M | DLBCL | R-CHOP, RB, Rm, Pola-BR | 3 | 219 | 7 | 5 | 1740 | >0.1 | 34647 |
| 27 | 81 | | M | MALT | R, Rm, RB, Len+R | 5 | 139 | 0 | 4 | 84.5 | 0.11 | 31406 |
| 28 | 54 | | F | FL | GB, Gm | 1 | 20 | 5 | None | 2153 | >0.1 | 619 |
| 29 | 72 | | M | FL | GB, Rm | 1 | 19 | 1 | None | 599 | >0.1 | 207 |
| 30 | 84 | | F | B-NHL | R-CHOP, RB, Pola-R-CHP | 3 | 89 | 2 | 5 | 437 | 0.14 | 1216143 |
| 31 | 63 | | F | DLBCL | VP-16, R-CHOP, GB, Pola-R-CHP | 4 | 126 | 3 | 4 | 635 | >0.1 | 3592833 |
| 32 | 84 | | F | DLBCL | R-CHOP, Pola-R-CHP | 2 | 57 | 1 | 4 | 2691 | >0.1 | 36750796 |
| 33 | 66 | | M | MCL | R-CHOP/R-DHAP, IBR, RB | 2 | 24 | 0 | 5 | 3839 | 6.69 | 49666880 |
| 34 | 81 | | M | DLBCL | Pola-R-CHP | 2 | 11 | 1 | 5 | 359 | >0.1 | 1476769 |
| 35 | 39 | | M | B-LBL | R-CHOP | 1 | 1 | 0 | 4 | 3043 | 0.38 | 1028 |
| 36 | 78 | | M | DLBCL | Pola-R-CHP | 1 | 8 | 3 | 5 | 1604 | >0.1 | 19879 |
| 37 | 81 | | F | DLBCL | R-CHOP, RB, Pola-BR | 1 | 14 | 7 | 2 | 424 | >0.1 | 950144 |
| 38 | 54 | | M | FL | GB, Rm | 1 | 16 | 3 | None | 55 | >0.1 | 31300 |
| 39 | 75 | | M | DLBCL | R-CHOP | 1 | 23 | 19 | 3 | 282 | 3.59 | 13929 |
| 40 | 64 | | F | DLBCL | R-CHOP, R-ESHAP, RB, Rm | 3 | 41 | 19 | 5 | 497 | 0.1 | 5505821 |
| 41 | 60 | | F | DLBCL | Pola-R-CHP | 1 | 2 | 0.5 | 4 | 101 | 0.72 | 4121 |
| 42 | 86 | | F | DLBCL | R-CHOP, R-EPOCH | 1 | 68 | 60 | 3 | 2.86 | 0.1 | 814250 |
| 43 | 60 | | F | FL | GB | 1 | 27 | 22 | None | 159 | 0.18 | 25843 |
| 44 | 49 | | M | FL | GB, Gm | 1 | 27 | 19 | 2 | 9.7 | >0.1 | 191 |
| Abbreviations: FL, follicular lymphoma; DLBCL, diffuse large B-cell lymphoma; LPL, lymphoplasmacytic lymphoma; MALT, mucosa-associated lymphoid tissue lymphoma; B-NHL, B-cell non Hodgkin lymphoma; MCL, mantle cell lymphoma; B-LBL, B-lymphoblastic lymphoma; GB, obinutuzumab+bendamustine; Gm, obinutuzumab maintenance; R-THP-COP, rituximab+pirarubicin+vincristine+cyclophosphamide+prednisolone; Rm, rituximab maintenance; R-CVP, rituximab+cyclophosphamide+vincristine+prednisolone; G-CHOP, obinutuzumab+cyclophosphamide+doxorubicin+vincristine+prednisolone; R-CHOP, rituximab+cyclophosphamide+doxorubicin+vincristine+prednisolone; RB, rituximab+bendamustine; DRC, dexamethasone+rituximab+cyclophosphamide; Pola-BR, polatuzumab vedotin+bendamustine+rituximab; R, rituximab; ASCT, autologous stem cell transplantation; Len+R, lenalidomide+rituximab; VP-16, etoposide; Pola-R-CHP, polatuzumab vedotin+rituximab+cyclophosphamide+doxorubicin+prednisolone; R-DHAP, rituximab+dexamethasone+cytarabine+cisplatin; IBR, ibrutinib; R-ESHAP, rituximab+etoposide+methylprednisolone+cytarabine+cisplatin; R-EPOCH, rituximab+etoposide+prednisolone+vincristine+cyclophosphamide+doxorubicin. | | | | | | | | | | | | |
