# Supplementary material for: The evaluation of risk factors for prolonged viral shedding during anti-SARS-CoV-2 monoclonal antibodies and long-term administration of antivirals in COVID-19 patients with B-cell lymphoma treated by anti-CD20 antibody
Source: BMC Infect Dis. 2024 Jul 22;24:715. doi: 10.1186/s12879-024-09631-3 (PMC11265166; doi:10.1186/s12879-024-09631-3)
Supplement: Supplementary file 2 — Supplementary Material 2. [file 12879_2024_9631_MOESM2_ESM.docx]

| **Supplementary Table 2. SARS-CoV-2 Subvariants, Treatment, and Severity of COVID-19** | | | | | | | | | |  |
| --- | --- | --- | --- | --- | --- | --- | --- | --- | --- | --- |
| No. | PANGOLIN | Monoclonal antibodies | | | Antivirals | Corticosteroid | Baricitinib | Severity of COVID-19 | Time from onset to viral shedding (days) | Nucleocapsid protein antibody at viral shedding (U/ml) |
|  |  | Sotrovimab | Casirivimab/ Imdevimab | Tixagevimab/ Cilgavimab | Antiviral  (days administered) |  |  |  |  |  |
| 1 | BA.1.1 | (+) |  |  | Rem (8) | (+) |  | Severe | 33 | >0.1 |
| 2 | BA.1.1.2 | (+) |  |  | Rem (10) | (+) |  | Moderate | 21 | 21.4 |
| 3 | BA.1.1 | (+) | (+) |  | Rem (7)→Nir (5)→**R**→Rem+Nir (8)→Mol (5)→**R**→Mol (10) | (+) | (+) | Severe | 104 | > 0.1 |
| 4 | BA.1.1.2 | (+) |  |  | Rem (10)→Nir (5) | (+) |  | Moderate | 25 | >0.1 |
| 5 | BA.2.3 |  | (+) |  | Rem (10) |  |  | Moderate | 27 | >0.1 |
| 6 | BA.1.1.1 | (+) | (+) |  | Rem (7)→Nir (5)→Rem+Nir (8)→Mol (5)→**R**→Mol |  |  | Moderate | 98 | >0.1 |
| 7 | BA.2.29 |  | (+) |  | Rem+Nir (5) |  |  | Moderate | 14 | >0.1 |
| 8 | BA.2.3 |  | (+) |  | Rem+Nir (4)→Nir (6) |  |  | Moderate | 41 | >0.1 |
| 9 | BA.2.3.13 |  | (+) |  | Rem (5)→**R**→Rem (10)→**T**→Mol (5) | (+) | (+) | Severe | 37 | >0.1 |
| 10 | BA.5.2.1 |  | (+) |  | Mol (5)→R→Mol (5) | (+) |  | Severe | 24 | >0.1 |
| 11 | BE.1.1 |  | (+) |  | Rem (5)→Mol (10) |  |  | Moderate | 18 | >0.1 |
| 12 | BA.5.1 |  | (+) |  | Rem (5)→Mol (10)→Nir (5)→Mol+Nir (10)→Rem (5)→Rem+Nir (5) |  |  | Moderate | 40 | >0.1 |
| 13 | BA.5.2.20 |  | (+) |  | Mol+Nir (10) |  |  | Moderate | 27 | >0.1 |
| 14 | BA.5.2 |  | (+) |  | Mol (10)→Nir (5) |  |  | Severe | 17 | 0.11 |
| 15 | BF.5 |  | (+) |  | Rem+Nir (5)→Mol (15) | (+) | (+) | Severe | 46 | >0.1 |
| 16 | NA |  | (+) |  | Mol (5)→**R**→Rem+Nir (5) | (+) | (+) | Severe | 34 | >0.1 |
| 17 | BA.5.2.1 |  | (+) |  | Mol (5)→**R**→Rem (8)→**T**→Rem+Nir (10)→Mol (5) | (+) | (+) | Severe | 76 | >0.1 |
| 18 | BA.5.1 |  | (+) | Prophylactic | Rem (30)→Nir (5)→**T**→Nir+Mol (15)→Mol (5) | (+) | (+) | Severe | 92 | 0.29 |
| 19 | BA.5.1 |  | (+) |  | Mol (5)→**R**→Mol+Nir (5) |  |  | Moderate | 17 | 14.8 |
| 20 | BF.7.15 |  | (+) |  | Mol (5) |  |  | Moderate | 7 | 0.1 |
| 21 | BA.5.2.1 |  | (+) |  | Mol+Nir (5) |  |  | Moderate | 11 | 0.45 |
| 22 | BU.1 |  | (+) |  | Rem (5)→Mol+Nir (5) | (+) |  | Moderate | 14 | 0.27 |
| 23 | NA |  |  | (+) | Mol (10) |  |  | Moderate | 25 | >0.1 |
| 24 | BA.5.1 |  |  | (+) | Mol+Mir (5) |  |  | Moderate | 33 | >0.1 |
| 25 | BF.5 |  |  | (+) | Rem+Ens (5)→Mol+Ens (10) |  |  | Moderate | 35 | >0.1 |
| 26 | BA.5.2.1 |  | (+) | Prophylactic | Rem (10)→Ens (10)→Nir (10)→Mol+Nir (5) | (+) |  | Critical | 33 | 0.33 |
| 27 | BA.5.2.1 |  | (+) | (+) | Rem (20)→T→Mol+Ens (10) | (+) |  | Critical | 185 | >0.1 |
| 28 | BA.5.2.1 | (+) |  | Prophylactic | Mol (3)→Rem (8)→**R**→Rem (10)→**R**→Rem (12)→**T**→Mol+Nir (5) | (+) |  | Severe | 129 | >0.1 |
| 29 | BA.5.2.7 | (+) |  | Prophylactic | Ens (5)→Mol (5) |  |  | Severe | 19 | >0.1 |
| 30 | FR.1 | (+) |  |  | Ens (10) |  |  | Moderate | 20 | 0.11 |
| 31 | FR.1 | (+) |  |  | Ens (5)→**R**→Rem (5) |  |  | Moderate | 30 | 58.4 |
| 32 | FR.1 | (+) |  |  | Rem (2)→Ens (5) |  |  | Moderate | 41 | 0.09 |
| 33 | FR.1 | (+) | (+) | Prophylactic | Mol (10)→Ens (5)→Rem+Nir (5)→Ens (10)→Mol+Nir (15)→Nir (5) | (+) |  | Severe | 57 | 49.2 |
| 34 | FR.1 | (+) |  |  | Ens (10)→R→Mol (5)→Ens (23) |  |  | Moderate | 34 | 0.68 |
| 35 | FR.1 | (+) |  |  | Ens (5)→Mol+Ens (5)→Nir (5) | (+) |  | Moderate | 27 | 146 |
| 36 | XBB.2.3.2 | (+) |  |  | Ens (10) |  |  | Severe | 24 | 0.2 |
| 37 | FR.1 | (+) |  |  | Ens (15) |  |  | Severe | 29 | >0.1 |
| 38 | XBB.1.16.1 | (+) |  |  | Ens (10)→Nir (10) |  |  | Moderate | 21 | >0.1 |
| 39 | XBB.1.16.2 | (+) |  |  | Ens (5) |  |  | Moderate | 16 | 109 |
| 40 | XBB.1.16.7 | (+) |  |  | Ens (10) |  |  | Moderate | 31 | > 0.1 |
| 41 | EG.1 | (+) |  |  | Ens (5) | (+) |  | Moderate | 7 | 0.54 |
| 42 | EG.5.1 | (+) |  |  | Ens (5) |  |  | Moderate | 14 | >0.1 |
| 43 | EG.5.1.6 | (+) |  | Prophylactic | Rem+Nir (5)→Nir (10) | (+) | (+) | Severe | 29 | 133 |
| 44 | BA.2.86.1 | (+) |  |  | Rem+Nir (5)→Nir (5) | (+) | (+) | Severe | 14 | 114 |
| Abbreviations: PANGOLIN, Phylogenetic Assignment of Named Global Outbreak Lineages; Rem, remdesivir; Nir, nirmatrelvir/ritonavir; **R**, rebound of viral load; Mol, molnupiravir; **T**, Transferred to our hospital; NA, not applicable; Ens, ensitrelvir. | | | | | | | | | | |
