# Supplementary material for: The evaluation of risk factors for prolonged viral shedding during anti-SARS-CoV-2 monoclonal antibodies and long-term administration of antivirals in COVID-19 patients with B-cell lymphoma treated by anti-CD20 antibody
Source: BMC Infect Dis. 2024 Jul 22;24:715. doi: 10.1186/s12879-024-09631-3 (PMC11265166; doi:10.1186/s12879-024-09631-3)
Supplement: Supplementary file 4 — Supplementary Material 4. [file 12879_2024_9631_MOESM4_ESM.docx]

| **Supplementary Table 4. Comparison of results from single nucleotide polymorphism PCR assays and genomic analysis.** |
| --- |

| No | Genomic analysis | Comparison of the results | Single nucleotide polymorphism PCR assays | | | | | |
| --- | --- | --- | --- | --- | --- | --- | --- | --- |
|  | PANGOLIN | Match or mismatch | Possible variants | E484K | L452R | ins214EPE | G339D | N460K |
| 1 | BA.1.1 |  | NA |  |  |  |  |  |
| 2 | BA.1.1.2 |  | NA |  |  |  |  |  |
| 3 | BA.1.1 |  | NA |  |  |  |  |  |
| 4 | BA.1.1.2 |  | NA |  |  |  |  |  |
| 5 | BA.2.3 | match | BA.2 |  | (-) | (-) |  |  |
| 6 | BA.1.1.1 | match | BA.1 |  | (-) | (+) |  |  |
| 7 | BA.2.29 | match | BA.2 |  | (-) | (-) |  |  |
| 8 | BA.2.3 | match | BA.2 |  | (-) | (-) |  |  |
| 9 | BA.2.3.13 | match | BA.2 |  | (-) | (-) |  |  |
| 10 | BA.5.2.1 | match | BA.5 |  | (+) |  | (+) |  |
| 11 | BE.1.1 | match | BA.5 |  | (+) |  | (+) |  |
| 12 | BA.5.1 | match | BA.5 |  | (+) |  | (+) |  |
| 13 | BA.5.2.20 | match | BA.5 |  | (+) |  | (+) |  |
| 14 | BA.5.2 | match | BA.5 |  | (+) |  | (+) |  |
| 15 | BF.5 | match | BA.5 |  | (+) |  | (+) |  |
| 16 | NA |  | BA.5 |  | (+) |  | (+) |  |
| 17 | BA.5.2.1 | match | BA.5 |  | (+) |  | (+) |  |
| 18 | BA.5.1 | match | BA.5 |  | (+) |  | (+) |  |
| 19 | BA.5.1 | match | BA.5 |  | (+) |  | (+) |  |
| 20 | BF.7.15 | match | BA.5 |  | (+) |  | (+) |  |
| 21 | BA.5.2.1 | match | BA.5 |  | (+) |  | (+) |  |
| 22 | BU.1 | match | BA.5 |  | (+) |  | (+) |  |
| 23 | NA |  | BA.5 |  | (+) |  | (+) | (-) |
| 24 | BA.5.1 | match | BA.5 |  | (+) |  | (+) | (-) |
| 25 | BF.5 | match | BA.5 |  | (+) |  | (+) | (-) |
| 26 | BA.5.2.1 | match | BA.5 |  | (+) |  | (+) | (-) |
| 27 | BA.5.2.1 | match | BA.5 |  | (+) |  | (+) | (-) |
| 28 | BA.5.2.1 | match | BA.5 |  | (+) |  | (+) | (-) |
| 29 | BA.5.2.7 | match | BA.5 |  | (+) |  | (+) | (-) |
| 30 | FR.1 | mismatch | XBB |  | (-) |  | (-/-) | (+) |
| 31 | FR.1 | mismatch | XBB |  | (-) |  | (-/-) | (+) |
| 32 | FR.1 | mismatch | XBB |  | (-) |  | (-/-) | (+) |
| 33 | FR.1 | mismatch | XBB |  | (-) |  | (-/-) | (+) |
| 34 | FR.1 | mismatch | XBB |  | (-) |  | (-/-) | (+) |
| 35 | FR.1 | mismatch | XBB |  | (-) |  | (-/-) | (+) |
| 36 | XBB.2.3.2 | match | XBB |  | (-) |  | (-/-) | (+) |
| 37 | FR.1 | mismatch | XBB |  | (-) |  | (-/-) | (+) |
| 38 | XBB.1.16.1 | match | XBB |  | (-) |  | (-/-) | (+) |
| 39 | XBB.1.16.2 |  | NA |  |  |  |  |  |
| 40 | XBB.1.16.7 | match | XBB |  | (-) |  | (-/-) | (+) |
| 41 | EG.1 | match | XBB | (-/-) | (-) |  |  |  |
| 42 | EG.5.1 | match | XBB | (-/-) | (-) |  |  |  |
| 43 | EG.5.1.6 |  | NA |  |  |  |  |  |
| 44 | BA.2.86.1 | match | BA.2.86 | (-/-) | (-/-) |  |  |  |

*PANGOLIN* Phylogenetic Assignment of Named Global Outbreak Lineages, *NA* not applicable, *(+)* positive specific mutation and negative wild type, *(-)* negative specific mutation and positive wild type, *(-/-)* negative specific mutation and negative wild type (This could mean that neither could be detected due to low viral titres or a different mutation.)
